# Albumanuria

**Published:** 1856-11

**Authors:** F. B. Norcum


### THE NORTH-WESTERN

### MEDICAL AND SURGICAL JOURNAL.

(NEW SERIES.)

VOL. V. NOVEMBER, 1856. NO. 11.

### ORIGINAL COMMUNICATIONS.

ARTICLE I.—*Albumanuria.* By F. B. NORCUM, M.D.

Wards 9 and 10.—John Welsh; aged 45 years; porter by occupation; native of Ireland; in this country 13 years, and admitted to Hospital Oct. 17th, 1854:—

Born of healthy parents, who still survive, and knows of no hereditary tendencies to disease existing in his family, or collateral branches. During his youth followed the sea for 9 years, and tho' exposed to many hardships, always enjoyed vigorous health. Has never suffered from any form of venereal disease, a circumstance which he ascribes more to his good fortune than to continence, having been addicted to excessive sexual indulgence, and often suffered great debility therefrom. Never drank to any excess until about six years ago, when he became exceedingly intemperate in his habits, consuming daily large quantities of various stimulating fluids.

Three years back, after a sudden and violent physical effort, was seized with great pain in his back, and soon after with frequent desire to pass his water. The secretion was high colored, scanty, and scalding; passed with extreme difficulty, and causing intense pain. Occasionally it would become more copious, of a lighter hue, and less acrid. Began now to experience attacks of headache, vertigo, and had considerable dyspnœa from any unusual exertion. About one year after the *apparent* commencement of this disease, his hands and feet began to swell, and he frequently noticed a puffiness of the lower lids, notwithstanding he continued to work, and follow the usual routine of irregular life, until a year ago last April, when he gave himself up to medical treatment. May, 1854, he had several attacks of hæmatemesis, vomiting, as he says, large clots of blood. In June, having considerably improved, he resumed his business, but after a trial of four or five days was obliged to discontinue, his legs and genital organs swelling enormously. He now remained at home until October, having in the meanwhile two severe epileptic attacks, and during his residence in hospital has had one such, remaining more or less insensible for 24 hours. Upon his admission, an examination of his urine revealed the nature of the disease; highly albuminous, of low specific gravity, and containing a sediment made up of fatty tube casts, degenerated renal epithelium, and occasionally blood corpuscles.

To relieve this dropsy, our treatment was addressed to the skin, kidneys, and alimentary canal, and with so much success that he became comparatively free from external appearance of disease, and quite sanguine of a radical cure. In April the swelling began again to increase, and abundant effusion took place in the abdominal cavity, causing distressing orthopnœa. During May it became so great that he could neither move or be moved except with the greatest caution, and his penis and scrotum invaded to such a degree that a fissure occurred in the dependant portion of latter, thro' which dribbed daily some two or three quarts of pale straw colored serum, having a strong *urinous* odor. Punctures were made in his lower extremities, and the drain so rapid that the swelling soon subsided, enabling him to locomote with comparative ease, while active purgation so far reduced the abdominal effusion as to free the breathing apparatus, and thus restore him to something of his usual comfort. He continued to enjoy this exemption from pain and extreme anasarca until early in August, when his condition began again to grow worse; his extremities to swell; pain in the lumbar region become troublesome; dyspnœa once more alarmingly intense; the stomach irritable, and the appetite poor and capricious. The urine normal in quantity, pale, of low sp. gr. loaded with albumen, and containing the microscopical elements above described. The patient now resisted the action of our remedies, and, notwithstanding every effort, began to fail; on the 28th, became somnolent; coma gradually supervened, and death occurred on the 30th.

### AUTOPSY, TWENTY-THREE HOURS AFTER DEATH.

*External Appearance.*—Body warm, pale, and anasarcous. Abdomen immensely distended.

*Thorax.* — Lungs compressed, and cavity diminished by upward pressure upon diaphragm. Right lung strongly adherent posteriorly and to diaphragm; tissue more dense than natural. Left lung slightly crepitant, approaching carnification, not adherent.

Heart and membranes healthy.

*Abdomen.*—Intestines blanched; stomach strongly adherent to transverse colon, and cavity of abdomen filled with four gallons of pale limpid serum. Mucous membrane of alimentary canal thickened and œdematous. Liver weighs 3½℔s. and presents a fine specimen of external cirrhosis; its peritoneal investment thickened and substance fatty. Kidneys small, weight 8oz. their external appearance smooth, pale, and mottled; capsule thickened, and so firmly adherent as to render it impossible to be stripped off without removing a portion of the substance of the organ. On section, the appearance was striking; cortical substance slightly diminished, but whitish, and contrasting strongly with the nearly healthy, well-defined, and reddish aspect of pyramids.

On microscopical examination, the tubes of cortical portion were generally coarsely granular, those of medullary also granular and fatty, and in some the walls were much thickened. The epithelium also granular, hypertrophied, fatty, and numerous tube casts were visible precisely similar to those observed in the urine during life.

The malpighion tufts were indistinct, as if blurred by adventitious plastic exudation; some inclosed in a thickened capsule.

A section of the matrix did not present any marked thickening.

The remaining viscera, not here noticed, were to all appearances normal, and presented no marked lesions.

This patient presents to us a well-marked example of "Bright's disease," known as the "granular degeneration" of Christison; the "albuminous nephritis" of Rayer & Lebert; or the "chronic, desquamative nephritis" of Johnson. Dr. Bright holds the opinion, that, primarily, it is a *functional* disorder; yet, a limited time after its inception, it has proceeded so far as to bid defiance to any form of treatment:* while M. Rayer, in his treatise, speaks of it as a *variety* or *modification* of inflammation. Many English physicians, and among them Dr. Christison, appear to recognize a state of acute congestion as utterly distinct from acute nephritis; the latter, as he says, being exceedingly rare in Scotland.

Dr. Robinson, however, takes a different view of this disease, and one towards which many pathologists are fast verging. He recognizes the similarity of symptoms between acute nephritis and the incipient stage of albuminous kidney, and, in his fourth chapter, asserts the identity of the causes between the two. Again, M. Lebert, from his researches, confirms the opinion of those who regard it as inflammatory;† and Dr. Johnson admits that acute nephritis passes *occasionally*, by almost imperceptible gradations, into the chronic, and from their immediate connection will be found mutually to illustrate each other.‡ Now, viewing the opinions of these authors, we perceive that many recognize the *essence* of this disease as acute inflammatory from the start, while others understand it as passive in its character, and to develop itself so slowly and insidiously, as to be unrecognizable until the attention is directed to it by the supervention of a congestive paroxysm, or some intercurrent complication. Our experience has been so limited, that it might seem supererogatory in us to hazard an opinion, yet, from a careful study of some half dozen cases under our immediate supervision, we can assert, that, in no instance did the disease commence as acute.

* Memoirs, 1827, 1831, 1836.

† Medico-Chirurgical Review, Jan. 1846.

‡ Op. Cit. page 168.

The patient whose case we have now under consideration, was seized suddenly, after some unusual exertion; yet the pain in the lumbar region, and the condition of the renal secretion, merely pointed to a congestive state of the kidneys, according to Christison utterly distinct from inflammation. Besides, the symptoms indicative of constitutional disturbance, and more or less present in all inflammatory attacks, were here wanting; the phenomena were purely local; and it was only after some months duration that any sympathetic irritation was manifested.

It was three years after this seizure, that the patient entered the hospital; and the examination of the urine revealed a disease well established and of long standing. If we look to the starting point of degeneration as a diseased condition of the blood, [Johnson,] induced probably in this case by the abuse of alcoholic liquors, who can tell at what time the disease commenced? or that it may not long have existed before the attention of the patient was directed to his condition? We do not mean to deny that Bright's kidney may not arise from an acute inflammatory disorder, but we desire to be ranked among those eclectic inquirers — not exclusionists — who regard this disease as passive, and view it as congestive, contra-distinguished from inflammatory.

"A deposit of oil in the denuded tubes is not an uncommon occurrence, but is generally viewed in only a few of them." "It also is seen at times within the cells, but more frequently in clustered masses." "The disintegrating process going on within the tubules, will explain the appearance of the granular amorphous masses in the urine." Presence of blood corpuscles (seldom met with in this advanced stage of the disease, an explanation offered by the thickened walls of the malpighion capillaries) was only occasional, and contemporaneous with the paroxysm of congestion; the result of rupture, and not of simple exosmose. The smooth, mottled, external surface of the kidneys; here pale, from decrease of vascularity—there dark, from engorgement; marks an advanced stage of the disease, as also the thickened and adherent capsule.

A section exhibits the cortical substance slightly diminished, its true texture taken up by the plastic deposit, and giving the white waxy-like appearance.

"The blurred condition of the malpighion tufts is evidently due to the presence of an organized material, either fibrous or cellular, within the capsule; presenting a thickened appearance, a very rare occurrence, inasmuch as being generally due to an increased thickness of the capillary walls, leading to a close packing and crowding of the vessels, as almost to obscure their outline."

The heart and its membranes were healthy, and exhibited none of those lesions which Bright asserts are found in 65 cases out of 100. M. Rayer says, that not more than one-fifth of his cases suffered from this complication, and thinks that the influence of renal disease on cardiac to be much exaggerated. He is of the opinion that the latter is oftener the primary disease; while others, though acknowledging that the two may exist coincidently, still recognize them as oftener connected by cause and effect. Adhesions of the lungs were formed, and a state of compression from mechanical pressure. These organs seldom escape derangement, but such complications are insidious in their progress, their symptoms being masked by constitutional cachexia.

The liver of small weight, its substance fatty, and presenting a beautiful specimen of cirrhosis, by far the most common condition. Being exposed to the influence of those causes which induce renal disease, it is probable that, in the large number of cases, the two are the result of a common cause, and that this abnormal condition of one tends to produce an analogous condition of the other, especially when viewed under the physiological law of vicarious discharge of function.

If we turn back to the history of this patient, we will find that he suffered from several epileptic seizures, and one whilst an inmate of the hospital. These complications are of the most serious character, and, connected with the cerebro-spinal functions, assume the form of coma or convulsions. In chronic cases, these manifestations approach gradually, but occur so frequently that the disease, according to some authorities, may be said to have a natural tendency to terminate in this manner, and are associated commonly with a scanty secretion of urine, often large quantities of urea being detected in the blood. The once-held theory, that the presence of this agent acted as the immediate cause of these nervous phenomena, through some poisoning property, is now generally repudiated by many pathologists. "Dr. Frerichs gives an entirely new explanation, and states that the symptoms are not immediately due to the presence of urea, but to the carbonate of ammonia, which results from its decomposition within the blood-vessels." This opinion he supports by experiment, and furthermore adds, that if, in a patient laboring under uralmic poisoning, we examine the expired air, it will contain carbonate of ammonia, the amount in proportion to the intensity of the symptoms.*

We have been unable to confirm this opinion by our own experiments. The injection of a solution of carb. ammon. in the circulating current, appears more conclusive; yet, when we come to consider the many who labor under a large accumulation of urea in the blood, unassociated with any morbid manifestation, and observe that in many of these cases there seems to be no chemical change, we must conclude that a link is wanting in the chain of reasoning by which he endeavors to support his views. He assumes the existence (though totally ignorant of the nature) of some peculiar fermenting agent, which accounts for decomposition in the one case, and its absence to explain the want of change in another; and, until this is cleared up, his observations will require repetition before they can be admitted among the established doctrines of pathology.†

* Die Bright'sche Nierenkrankheit, pp. 107—112.

† Johnson, Op. Cit. p. 204.

